# Differential regulation of mitochondrial uncoupling protein 2 in cancer cells

**DOI:** 10.1016/j.bbabio.2024.149486

**Published:** 2024-07-08

**Authors:** Taraneh Beikbaghban, Ludovica Proietti, Jessica Ebner, Roko Sango, Thomas Rattei, Thomas Weichhart, Florian Grebien, Felix Sternberg, Elena E. Pohl

**Affiliations:** a Physiology and Biophysics, Department of Biological Sciences and Pathobiology, University of Veterinary Medicine, Vienna, Austria; b Institute for Medical Biochemistry, University of Veterinary Medicine, Vienna, Austria; c Centre for Microbiology and Environmental Systems Science, University of Vienna, Vienna, Austria; d Center of Pathobiochemistry and Genetics, Institute of Medical Genetics, Medical University of Vienna, Vienna, Austria; e Doctoral School in Microbiology and Environmental Science, University of Vienna, Vienna, Austria; f Center of Pathobiochemistry and Genetics, Institute of Medical Genetics, Medical University of Vienna, Austria; g St. Anna Children’s Cancer Research Institute (CCRI), Vienna, Austria; h CeMM Research Center for Molecular Medicine of the Austrian Academy of Sciences, Vienna, Austria; i Department of Nutritional Sciences, Faculty of Life Sciences, University of Vienna, Austria

**Keywords:** Warburg effect, Oxygen consumption rate, Extracellular acidification rate, Uncouplers, Metabolite transport, Citric acid cycle, Pyruvate kinase

## Abstract

The persistent growth of cancer cells is underscored by complex metabolic reprogramming, with mitochondria playing a key role in the transition to aerobic glycolysis and representing new therapeutic targets. Mitochondrial uncoupling protein 2 (UCP2) has attracted interest because of its abundance in rapidly proliferating cells, including cancer cells, and its involvement in cellular metabolism. However, the specific contributions of UCP2 to cancer biology remain poorly defined. Our investigation of UCP2 expression in various human and mouse cancer cell lines aimed to elucidate its links to metabolic states, proliferation, and adaptation to environmental stresses such as hypoxia and nutrient deprivation. We observed significant variability in UCP2 expression across cancer types, with no direct correlation to their metabolic activity or proliferation rates. UCP2 abundance was also differentially affected by nutrient availability in different cancer cells, but UCP2 was generally down-regulated under hypoxia. These findings challenge the notion that UCP2 is a marker of malignant potential and suggest its more complex involvement in the metabolic landscape of cancer.

## 1 Introduction

Cancer is one of the most common causes of death worldwide, with millions of deaths each year attributable to the various forms of cancer [1]. Despite the development of many novel therapeutics, cancer remains a treacherous disease due to the high metastatic potential of malignant cells and the emergence of multidrug resistance. Reprogramming of cellular energy metabolism has been identified as a hallmark of cancer [2,3]. While most cells rely on oxidative phosphorylation under physiological conditions, cancer cells shift their metabolism towards aerobic glycolysis [4,5]. Cancer cells reprogram their metabolism by using nutrients to fuel the increased biomass production required for uncontrolled growth and proliferation. To maximize their anabolic metabolism rather than ATP production, they maintain a high rate of glycolysis even in oxygen-rich conditions, a phenomenon known as the Warburg effect. The rapid proliferation of cancer cells often leads to a glucose shortage, which should limit their survival by constraining aerobic glycolysis. However, malignant cells can circumvent this limitation by mitochondrial metabolic shifts, thereby sustaining their metabolic needs under glucose-deprived conditions [6].

Uncoupling protein 2 (UCP2) is a member of the uncoupling protein subfamily in the mitochondrial anion carrier protein superfamily SLC25. UCP2 was identified in 1997 [7] by cDNA library screening for candidate genes that were homologous to uncoupling protein 1 (UCP1), which is responsible for non-shivering thermogenesis in brown adipose tissue [8]. UCP2 is localized at the inner mitochondrial membrane and has been implicated in transport of protons because its expression in yeast decreased the mitochondrial potential [7]. Proton transport in the presence of fatty acids was later demonstrated in well-defined artificial systems such as proteoliposomes and bilayer membranes reconstituted with the purified recombinant protein [9,10]. In addition to its proton-transporting function, more recent evidence revealed that UCP2 transports C4 metabolites, such as malate, oxaloacetate, and aspartate from the mitochondria to the cytosol [11–13].

However, the biological function of UCP2 is still under debate [14]. One major limitation of studies investigating UCP2 protein levels is the low specificity of commercial anti-UCP2 antibodies and the lack of antibody evaluation data in the studies presented. Studies with thouroughly evaluated antibodies have demonstrated the high abundance of UCP2 in pluripotent stem cells, immune cells and cancer cells [15–17]. These cells exhibit aerobic glycolysis as a main type of the metabolism and a high rate of proliferation.

Here, we hypothesize that upregulation of UCP2 leads to metabolic flexibility of cancer cells during nutrient deprivation, which drives glycolysis after conversion to pyruvate in the cytosol. Therefore, we aimed (i) to analyze UCP2 protein levels in different cancer cell lines and correlate it with glycolytic metabolism; (ii) to investigate UCP2 changes under challenging conditions such as hypoxia or glutamine or glucose deprivation; and (iii) to evaluate the correlation between UCP2 abundance and cell proliferation. To this end, we performed real-time cell metabolism analysis, Western blotting and proliferation assays on several murine and human cancer cell lines, including CRISPR-Cas-9 generated UCP2 KO cell lines.

## 2 Materials and methods

### 2.1 Cell lines and culture conditions

Human acute myeloid leukemia (AML) cell lines, including HL-60 (ACC 3), MOLM-13 (ACC 554), MV4-11 (ACC 102), THP-1 (ACC 16) were acquired from DSMZ (Deutsche Sammlung von Mikroorganismen und Zellkulturen GmbH, Braunschweig, Germany). Each cell line was authenticated using STR profiling. Our study also included N18TG2 mouse neuroblastoma cells (DSMZ) and MC38 mouse colon adenocarcinoma cells.

AML cell lines (HL-60, MOLM-13, MV4–11, and THP-1) and the K562 chronic myeloid leukemia (CML) cell line were maintained in RPMI 1640 (2522621, Gibco, USA) medium enriched with GlutaMAX™ (2,323,475, Gibco, China), supplemented with 10 % heat-inactivated fetal bovine serum (HI FBS), and 1 % penicillin/streptomycin. N18TG2 and MC38 cells were cultured in high glucose DMEM (2,436,844, Gibco, UK) with GlutaMAX™, supplemented with sodium pyruvate, 10 % HI FBS, and 1 % penicillin/streptomycin (15,140,122, Gibco, USA). All cell lines were incubated in a humidified atmosphere with 5 % CO_2_ at 37 °C. For nutrition shortage and hypoxic experiments, cells were seeded on poly-D-lysine (PDL1) precoated 6-well plates (Greiner Bio-One). Notably, we maintained pyruvate in the culture media during nutrient starvation to maintain basal cellular function and to dampen severe stress responses such as autophagy [53]. Following overnight incubation at 37 °C in a 5 % CO_2_ incubator, cells were washed twice with Dulbecco’s phosphate-buffered saline (dPBS). The medium was then replaced with complete DMEM, supplemented with 100 μM cobalt (II) chloride (CoCl2) (C8661, Sigma-Aldrich, USA) to induce hypoxic conditions for the indicated time points.

### 2.2 Protein isolation and Western blotting

Protein isolation, analysis and detection were performed according to [16]. In brief, the cells were washed in PBS, collected in a RIPA-buffer with a 1:50 protease inhibitor cocktail (#P8340, Sigma-Aldrich, Austria) together with PhosSTOPTM (#59124600, Roche, Germany), and sonicated for 3 s on ice. Total cellular protein was isolated after centrifugation and quantitatively determined using a Pierce BCA Protein Assay Kit (#RG235622, Thermo Fisher Scientific, Waltham, MA, USA). We loaded 20, 30 or 40 μg total cellular protein per lane as indicated in figure legends. We used antibodies against UCP2 evaluated previously ([15], Suppl. Fig. 1A and B). Antibodies against 60-kDa heat shock protein (HSP60, Santa Cruz Biotechnology, Texas, USA, sc-365,344, 1:1000), oxoglutarate dehydrogenase (OGDH, Proteintech, Manchester, UK, 15212-1-AP, 1:5000) and β-actin (Sigma-Aldrich; A5441; 1:5000) were used. Immunoreactions were performed by luminescence using a secondary antibody against rabbit or mouse antibodies linked with horseradish peroxidase (GE Healthcare, Austria) and ECL Western blotting reagent (Bio-Rad, Austria). The intensity of the bands was detected using the ChemiDoc-It 600 Imaging System (UVP, UK) and measured with Launch Vision Works LS software (UVP, UK). The immunoblot intensities of the different detected proteins were calculated as a ratio relative to the intensity of OGDH.

### 2.3 Metabolic analysis

K562, MC38 and N18TG2 cells were seeded (20,000 cells per well in control DMEM medium) on poly-D-lysine (A3890401, Gibco, USA) coated Seahorse 96XFe plates (103794–100, Agilent, CA, USA) for 16 h prior to the analysis. For the nutrient shortage experiments, cells were washed twice and filled with fresh corresponding medium (control or nutrient shortage medium). One hour before the experiment, the medium was changed to HEPES buffered XF base medium (12,923,001, Agilent, USA) containing the indicated amounts of glucose, glutamine, and 2 mM pyruvate, followed by incubation in a non-CO_2_ incubator for one hour before the experiment. Of note, the omission of FBS 1 h prior to Seahorse measurement is a prerequisite for the measurement of the extracellular acidification rate (ECAR), as recommended by the supplier (Agilent, CA, USA) and reported in numerous publications ([51,52]). The oxygen consumption rate (OCR) and ECAR were concomitantly measured using an XFe96 extracellular flux analyzer (S7850A, Seahorse Bioscience, Agilent, USA). The reagents of the Seahorse XF Cell Mito Stress Test Kit (103015–100, Agilent, USA) were applied according to the manufacturer’s instructions at the adjusted concentrations (1 μM (MC38 and N18TG2) or 2.6 μM (K562) oligomycin, 1.2 μM FCCP, 1 μM rotenone and 1 μM antimycin A, if not otherwise indicated). Each independent experiment was performed on a new plate, and on a separate day. Wells showing a non-homogeneous distribution in the cell layer were excluded from the analysis. The minimum number of wells per condition for each experiment was five (for the basal measurements) and three (Mito Stress Test, 103,015–100, Agilent, USA). The OCR and ECAR of each well were normalized to the μg protein content of the corresponding well, as determined by the Pierce BCA Protein Assay Kit (#RG235622, Thermo Fisher Scientific, Waltham, MA, USA), with a scaling factor of 100.

### 2.4 Proliferation assay

85,000 N18TG2, 85,000 MC38 or 350,000 K562 cells were seeded into 6-well microplates the day before the experiment. After overnight incubation at 37 °C, the medium was changed to the appropriate (control, starved, or hypoxic) medium and the plates were incubated and scanned for 96 h in the Incucyte® S3 Live-Cell Analysis System (Sartorius AG, Göttingen, Germany). Scanning was performed using a 20× objective. During the 72 h scan, 25 phase contrast images were captured per well in a two hour interval. Scans were analyzed and quantified by Incucyte® software (Sartorius AG, Göttingen, Germany). We calculated the time required for each cell line to double its cell number by defining the corresponding time points when the cells reached 20 % (T1) and 40 % (T2) of their confluence and subtracting them (ΔT), and presented it as the doubling time (Suppl. Fig. 1E). Data represent the mean of three technical replicates over the corresponding time window.

### 2.5 CRISPR-Cas9 mediated UCP2 knock-out

MOLM-13, HL60 and K-562 cells stably expressing Cas9 were cultured in RPMI-1640 medium (#2436534, Gibco, UK) supplemented with 10 % FBS, 100 U/mL penicillin, 100 μg/mL streptomycin and 2 mM L-glutamine (25,030,081, Gibco, Brazil). Lenti-X cells for virus production were cultured in DMEM (2,436,844, Gibco, UK), supplemented with 10 % FBS, 100 U/mL penicillin, 100 μg/mL streptomycin and 4 mM l-glutamine. All cells were kept at 37 °C with 5 % CO_2_ and routinely tested for contaminants.

SgRNAs targeting UCP2 were designed using the VBC score (vbc-score.org) [18,19] and cloned into a *Bsm*BI-digested lentiviral expression vector coupled to iRFP670 (pLenti-hU6-sgRNA-iRFP670). The sgRNA sequences used and the control sgRNA targeting *AAVS1* and *RPL17* are listed below:

sgUCP2 #1 GGAGCATGGTAAGGGCACAG,

sgUCP2 #2 GAATGGTGCCCATCACACCG,

sgAAVS1 GCTCCGGAAAGAGCATCCT,

sgRPL17 GTACCATTCCGACGTTACAA.

For virus production, semiconfluent Lenti-X cells were co-transfected with 1 μg pMD2.G (Addgene #122259), 2 μg psPAX2 (Addgene #12260) and 4 μg transfer vector using polyethylenimine (PEI). Lentiviral supernatants were collected 48 h after transfection, filtered with 0.45 μm filters and stored at 4 °C. Target cells were infected using 1:8 virus dilution in the presence of polybrene (10 μg/mL) (TR-1003-G, Merck, Billerica, MA, USA) and spinoculated for 90 min at 900 g at room temperature.

K-562 cells stably expressing Cas9 were infected with lentiviral vectors resulting in sgUCP2-iRFP670 expression. sgAAVS1 and sgRPL17 were used as negative and positive controls, respectively. The ratio of iRFP670-positive cells was assessed 3 days after spinoculation and measured at regular intervals by flow cytometry. Values were normalized to day 3 post-infection. Cytometric measurements were performed using IntelliCyt® IQue Screeener Plus (BioScience, Sartorius Group).

### 2.6 Untargeted RNA sequencing

Total RNA was extracted from K562 UCP2 wt and UCP2KO cells using the [Monarch Total RNA Miniprep Kit] (NEB #T2010S Monarch, MA, USA) according to the manufacturer’s instructions. The quality and concentration of RNA were assessed using [2100 Bioanalyzer/4200 TapeStation Agilent CA, USA]. Only samples with an RNA Integrity Number (RIN) above 7.0 were used for subsequent analysis.

Messenger RNA was purified from total RNA using poly-T oligoattached magnetic beads. After fragmentation, the first strand cDNA was synthesized using random hexamer primers followed by second strand cDNA synthesis. To create the libraries, A-tailing, adapter ligation, size selection, amplification, and purification was performed. The library integrity was evaluated with Qubit and real-time PCR; and bioanalyzer was used for size distribution detection. Quantified libraries were pooled and sequenced on Novaseq X plus, using PE150 sequencing strategy.

### 2.7 RNA-seq analysis

Raw reads were analyzed with a Nextflow v23.10.1 [20] workflow containing following steps: analysis of read quality, alignment of the reads and transcript quantification. The reads quality was verified by FastQC v0.12.1 (https://www.bioinformatics.babraham.ac.uk/projects/fastqc/) with default parameters, followed by index building and read alignment against GRCh38 reference genome with STAR v2.7.11 [21] and using Genode release 44 GTF file as an annotation parameter. For alignment with STAR the following parameters were specified: –outSAMtype BAM SortedByCoordinate –outSAMunmapped Within. Next, an indexed transcriptome was built with Gencode Human reference release 44, followed by quantification of transcripts. Here, indexing and quantifying was done with Salmon v1.10.3 [22]. Default parameters were used for index building, whereas for quantification following parameters were invoked: –gcBias –validateMappings. All reference datasets were obtained from https://www.gencodegenes.org/human/release_44.html.

QC and mapping quality information has been aggregated with MultiQC v1.19 [23], with report generated available at https://github.com/rsango6/VetMedUni_Project.

In R statistical environment v4.3.1 (https://www.r-project.org/), downstream differential gene expression analysis was performed with DESeq2 v1.40.2 [24]. Here we followed standard DESeq2’s normalization strategy (size factor calculation with median of ratios method). Moreover, the ‘independentFiltering’ parameter was set to TRUE, the alpha value was set to 0.05 and the *p*-value adjustment method was set to “Benjamini-Hochberg”. For shrinking Log2FoldChanges of noisy genes we opted for ashr adaptive shrinkage estimator, v2.2 [25]. For visualization, i.e. dimensionality reduction purposes the dataset was transformed with vst (variance stabilizing transformation) using vsn v3.68.0 [26].

For gene set overlap analysis, we opted for enrichR v3.2 [27], and a 2021 KEGG H.sapiens database [28]. Enriched gene sets were tested for significance with Fisher’s exact test at alpha value 0.05, corrected with Benjamini-Hochberg procedure. Visualization schemes were obtained with ggplot2 v3.4.2 [29].

## 3 Results

### 3.1 UCP2 basal levels are different among various cancers

To address the hypothesis that UCP2 can be considered as hallmark of malignancy, we investigated whether there was a correlation between UCP2 abundance and proliferation rates and metabolic activity. We analyzed UCP2 protein levels in several cancer cell models: human chronic myeloid leukemia (K562), human acute monocytic leukemia (THP1), human acute promyelocytic leukemia (HL60), human acute myeloid leukemia (MOLM13), human acute monocytic leukemia (MV4–11), mouse colon carcinoma (MC38), and mouse neuroblastoma (N18TG2) cells.

**Fig. 1:**
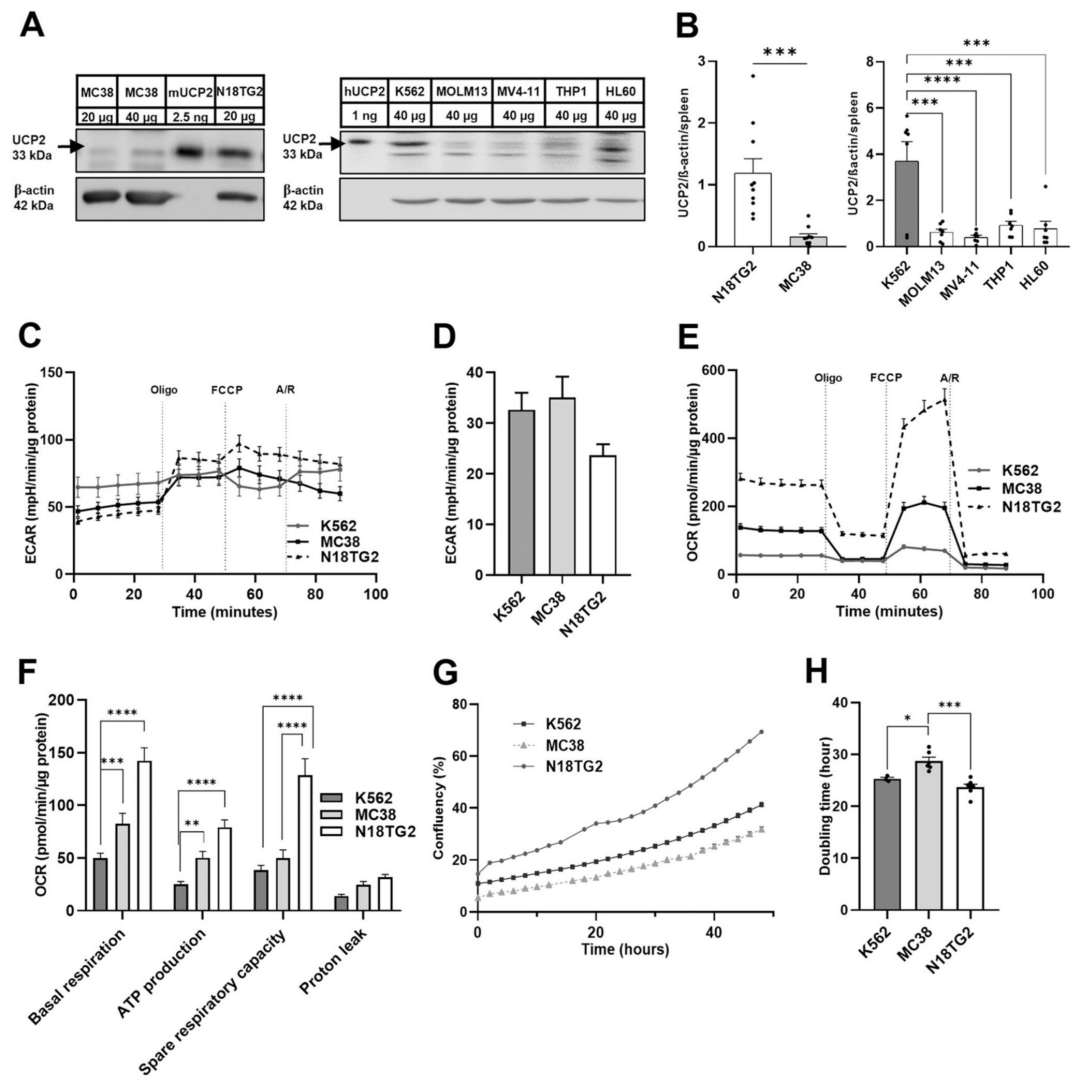
UCP2 expression, proliferation rate, OCR and ECAR levels vary significantly between human and murine cancer cell lines. (A) Representative Western blots of UCP2 expression in human and murine cell lines. (B) Quantification of UCP2 levels in mouse (MC38 and N18TG2) and human cancer cell lines (K562, MOLM13, MV4–11, THP1 and HL60). UCP2 protein levels were normalized to ß-actin levels and 20 μg mouse spleen were used as an internal control. Data show mean ± SEM from ten (N18TG2 and MC38) and seven (K562, MOLM13, MV4–11, THP1 and HL60) independent experiments. (C–D) Representative extracellular acidification rate (ECAR) of K562, MC38 and N18TG2 cells during a mito stress test and the quantitative basal ECAR analysis mean ± SEM from four independent experiments. (E) Representative measurements of oxygen consumption rate (OCR) in K562, MC38 and N18TG2 cells during a mito stress test. (F) Quantification of the basal OCR, ATP-linked OCR, spare capacity and proton leak in K562, MC38 and N18TG2 cells. Data show mean ± SEM from four independent experiments (C–F). Oligo, oligomycin; A/R, antimycin and rotenone. (G) Representative proliferation assay of K562, MC38 and N18TG2 cells for 48 h growth. (H) Time required for K562, N18TG2 and MC38 cells to reach double confluence (20 % to 40 %). Data show mean ± SEM from three (K562) or six (MC38 and N18TG2) independent experiments.

Fig. 1A shows a representative Western blot, comparing basal levels of UCP2 in these cell lines. Recombinant UCP2 and murine spleen extract were included as positive controls while murine spleen tissue from *Ucp2*-knock-out mice [17] was used as negative control (Fig. 1, Suppl. Fig. 1A and B). Although equal amounts of the total protein (40 μg) were loaded, the cancer cell lines showed large differences in UCP2 abundance with the highest amount in K562, followed by N18TG2, THP1, HL60, MOLM13, MV4–11 and MC38 cells (Fig. 1B and Suppl. Fig. 1C and D). From here we focused on K562, N18TG2 and MC38 as representative cell lines with very high, medium and very low UCP2 levels. Compared to solid tumors, leukemia cells are reported to be more strongly influenced by their environment and metabolism [30] [31]. Besides the critical role of the bone marrow microenvironment with factors such as bone morphogenetic proteins (BMPs) crucial for proliferation and survival [32], metabolic adaptations are key to promoting leukemia cell growth [33,34].

Therefore, to investigate whether the observed levels of UCP2 correlate with the metabolic state of the cancer cells, we compared the real-time extracellular acidification rate (ECAR, a measure of lactate deprotonation) in K562, N18TG2 and MC38 cells at basal levels and after the addition of mitochondrial respiratory complex inhibitors. As shown in (Fig. 1C, D), there was no clear correlation between UCP2 levels and glycolytic activity in these cells.

We further tested whether there was a correlation between UCP2 abundance and mitochondrial respiration by measuring basal OCR for the same cell lines. N18TG2 cells with moderate UCP2 expression showed the highest basal OCR (Fig. 1E and F). In contrast, K562 cells with the highest UCP2 levels of all cell lines analyzed had the lowest basal OCR (Fig. 1E and F). Mitochondrial ATP production, spare respiratory capacity and proton leak were consistent with basal respiration data (Fig. 1F). Finally, we analyzed cell proliferation rates of K562, N18TG2 and MC38 cell lines. K562 and N18TG2 cells showed higher proliferation and significantly shorter doubling times compared to MC38 cells (Fig. 1G and H).

Overall, although the relationship between UCP2 levels and proliferation rate appears to be more complex, the results indicated a trend towards an inverse correlation between UCP2 levels and oxidative respiration. Oxidative respiration, glycolytic response to oligomycinmediated inhibition of ATP synthase, and proliferation rates of N18TG2 were high while UCP2 levels were moderate. In contrast, K562 showed low oxidative respiration, no ECAR response to oligomycin, moderate proliferation and high abundance of UCP2. MC38 showed the lowest UCP2 levels, the longest doubling time, thus the slowest proliferation, and an intermediate OCR.

### 3.2 Variable effect of glucose and glutamine shortage on UCP2 levels in cancer cell lines

**Fig. 2:**
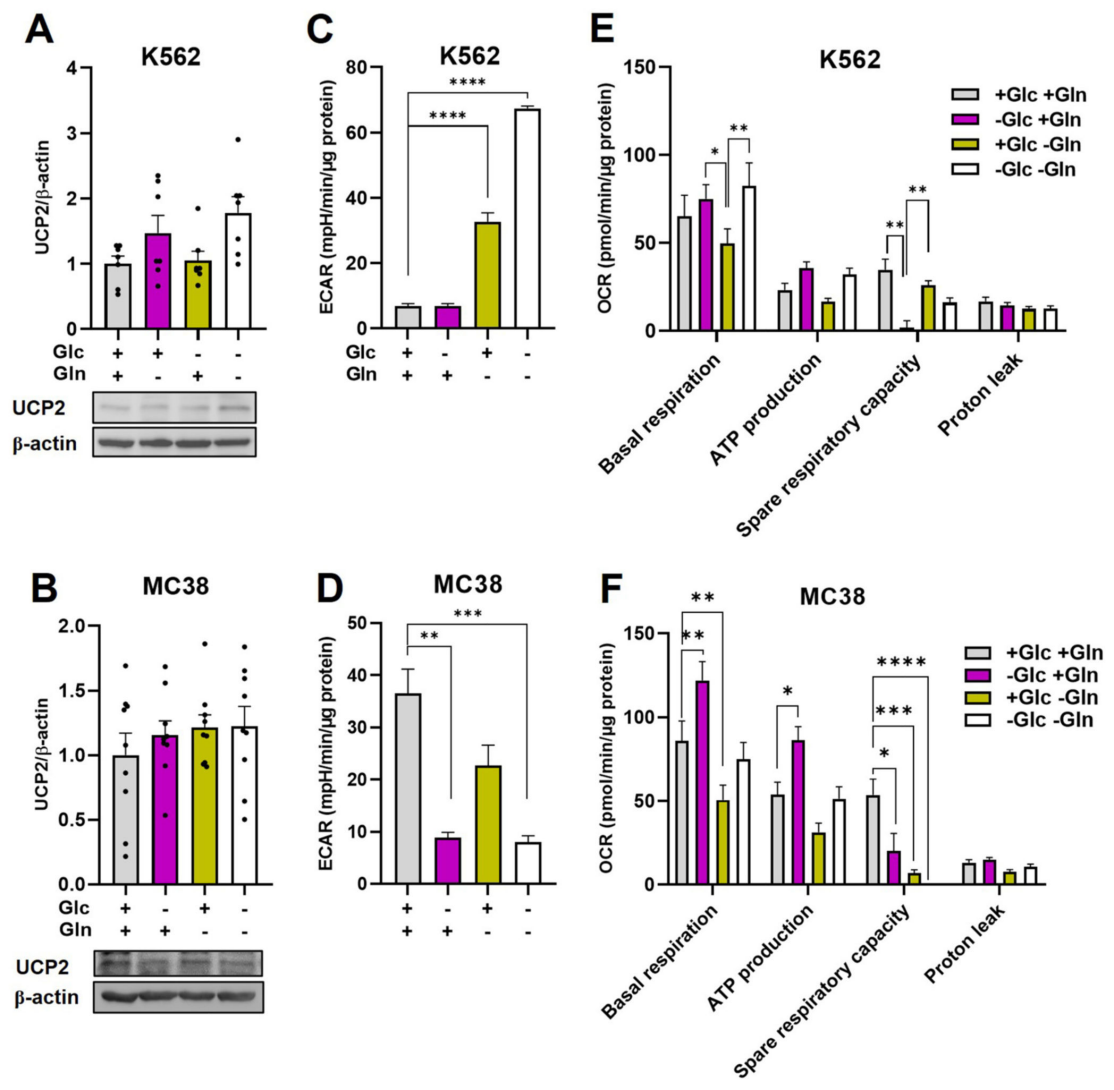
Differential changes in UCP2 levels under nutrient deprivation in different cell lines (A) and (B) UCP2 levels in K562 (A) and MC38 (B) cells after 4 h of deprivation of glucose, glutamine, both glucose (Glc) and glutamine (Gln) deprivation or control conditions (5.5 mM Glc, 2 mM Gln). The amount of protein is presented as the ratio between the band intensities of UCP2 and ß-actin and normalized to the band intensity of cells grown under control conditions (control is set to one). Data show mean values ± SEM of seven (A) and eight (B) independent experiments. (C–F) Effect of nutrient deprivation on the extracellular acidification rate (ECAR) in K562 (C) and MC38 (D) cells. Effect of nutrient deprivation on the oxygen consumption rate (OCR) in K562 (E) and MC38 (F) cells. Data show mean ± SEM of four independent experiments.

It was shown in N18TG2 cells that glucose (Glc) deprivation stimulates UCP2 upregulation, whereas glutamine (Gln) deprivation led to its downregulation [16]. To test whether similar trends in UCP2 abundance are consistent in various cancer cell types under nutrient stress, K562, MC38 and N18TG2 cells were subjected to different kind of nutrient deprivation, and UCP2 protein levels were analyzed. Surprisingly, neither K562 nor MC38 cells showed any significant changes in UCP2 levels under Gln or Glc starvation (Fig. 2A and B), despite its significant effect on metabolic flux: Gln starvation significantly increased ECAR in K562 cells (Fig. 2C), while significantly decreasing it in MC38 (Fig. 2D). In contrast, Gln starvation similarly decreased OCR in both cell lines. Glc deprivation had no effect on ECAR in K562 but repressed ECAR in MC38 (Fig. 2C and D). In addition, it increased and exhausted OCR in both K562 and MC38 cells to maximal respiratory capacity levels (Fig. 2E and F). We observed a significant downregulation of UCP2 in N18TG2 cells in response to Gln, which corresponded to an increase in OCR in N18TG2 cells (Suppl. Fig. 2A -C) that was consistent with published results [16].

To address the question of whether UCP2 abundance depends on glutamine concentration, we compared glutamine-depleted N18TG2, K562 and MC38 cells (1 μM Gln) with control cells (2 μM Gln) and cells under high glutamine concentrations (4 μM Gln). UCP2 expression directly correlated with Gln concentration in N18TG2 cells (Suppl. Fig. 3A and [16]), but this was not the case in either K562 (Suppl. Fig. 3B) or MC38 cells (Suppl. Fig. 3C). We then monitored cancer cell proliferation under different glutamine concentrations (Suppl. Fig. 3D-F). In contrast to N18TG2 (Suppl. Fig. 3D), the much slower proliferating MC38 cells increased proliferation in a exclusively glutamine-dependent mode (Suppl. Fig. 3C).

### 3.3 Downregulation of UCP2 in response to hypoxic conditions

**Fig. 3:**
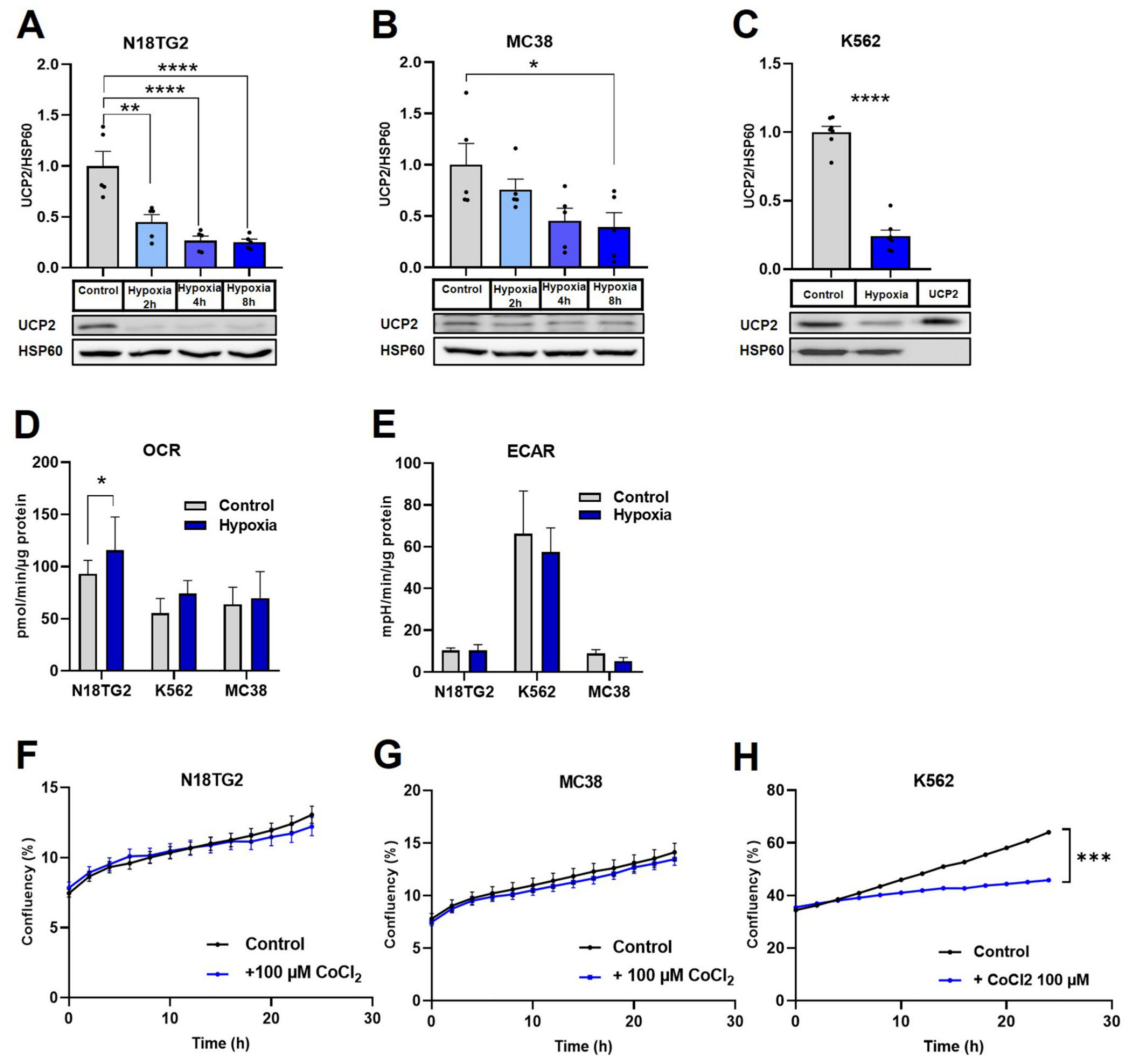
Changes in UCP2 expression, OCR, ECAR and cell proliferation under hypoxia-like conditions. (A-B) Representative Western blot and subsequent quantification of UCP2 protein levels in N18TG2 (A) and MC38 (B) cells, after 1, 4 and 8 h and in K562 after overnight incubation in 100 μM CoCl_2_ supplemented medium compared to control. The amount of protein is shown as the ratio between the band intensities of UCP2 and HSP60 and normalized to the band intensity of cells grown under control conditions (control is set to one). Figures show mean values ± SEM of five (A and B) or six (C) independent experiments. (D) Changes in oxygen consumption rate (OCR) and (E) extracellular acidification (ECAR) changes after 4 h of CoCl2 application compared to control in N18TG2, K562 and MC38 cells. Data show mean values ± SEM of three independent experiments. (F–H) Changes in proliferation rate under hypoxic conditions compared to control conditions in N18TG2 (F), MC38 (G) and K562 (H) cells.

Since proliferation and nutrient deprivation could not explain the variation in UCP2 levels in our cancer cell lines, we hypothesized that UCP2 levels may be altered under hypoxic conditions typical of the tumor environment. The sensitivity of UCP2 to hypoxia was already reported in different models [35,36]. Hypoxia, classically induced by low O_2_ conditions, is known to activate a number of cellular adaptations in cancer cells, including epithelial-to-mesenchymal transition (EMT) and metabolic reprogramming, which collectively contribute to metastasis and treatment resistance [37]. To evaluate UCP2 levels under hypoxic conditions, we subjected the N18TG2, K562, and MC38 cell lines to hypoxia-mimicking conditions using cobalt chloride (CoCl_2_), which is known to stabilize hypoxia-inducible factor 1-alpha (HIF1α). As shown in Fig. 3A and B we observed UCP2 downregulation at 2, 4, and 8 h after CoCl_2_ treatment in N18TG2 and MC38 cells. UCP2 protein analysis of K562 cells after overnight CoCl_2_ treatment showed similar findings (Fig. 3C).

We further investigated the correlation between UCP2 levels and metabolic pathways under hypoxic conditions by measuring ECAR and OCR in all three cell lines after 4 h of hypoxia. As shown in Figs. 3C and D, both OCR and ECAR remained not significantly changed after 4 h exposure to CoCl_2_ with the exception of modestly increased OCR in N18TG2 cells (Fig. 3D). The results rule out that UCP2 is decreased consequently to metabolic changes in MC38 cells and show that a 4-h exposure to CoCl2 is too short to induce significant metabolic changes in MC38 and K562 cells.

Finally, we investigated whether the decrease of UCP2 under hypoxic conditions correlated with changes in cell proliferation. Real-time proliferation assays of N18TG2, K562, and MC38 cells under CoCl2-induced hypoxia and normoxic conditions over 24 h showed significant decreased proliferation rates of K562 cells, but not N18TG2 and MC38 cells (Fig. 3F, G and H). Comparing UCP2 levels after long-term exposure of K562 (Fig. 3C) to CoCl_2_ and proliferation recording (Fig. 3H), results show a correlation in decreased UCP2 and decreased proliferation upon hypoxia-mimicking conditions, suggesting that these cancer cells depend on UCP2 under hypoxia.

### 3.4 Cell proliferation and metabolism remained unchanged in K562 UCP2KO cells

**Fig. 4:**
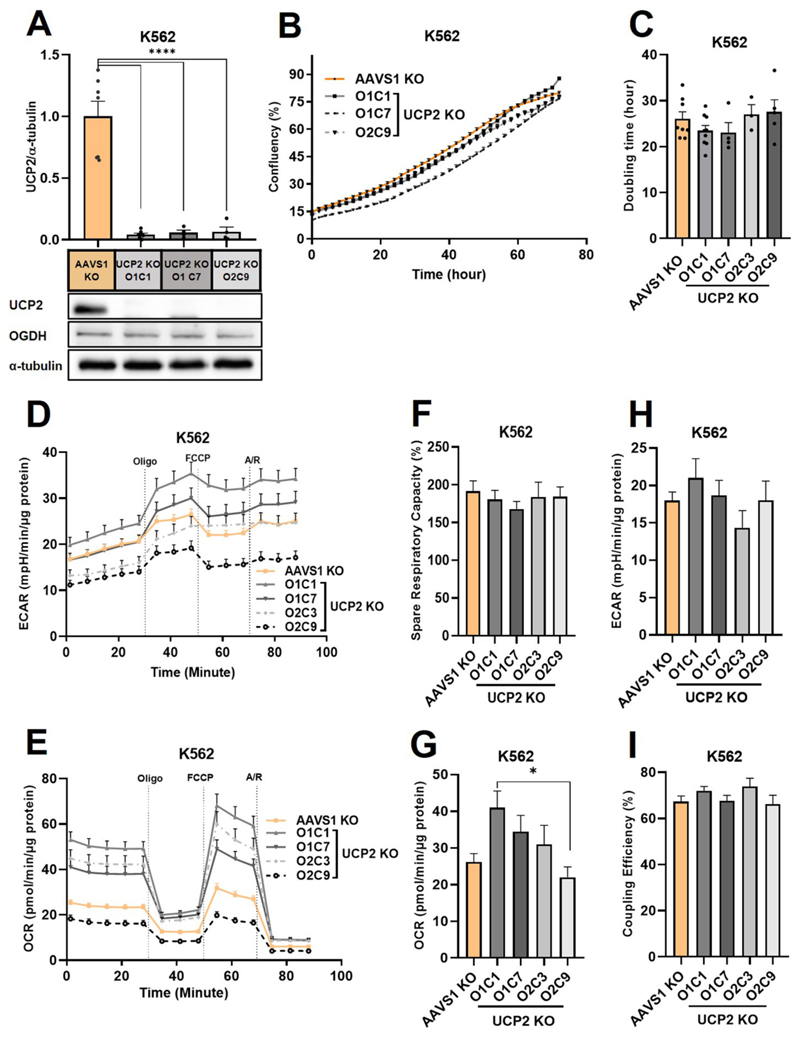
Effect of UCP2 knockout on proliferation rate and cell metabolism (A) UCP2 KO validation in K562 UCP2 KO single cell colonies compared to K562 AAVS1 KO. The amount of protein is presented as the ratio between the band intensities of UCP2 and α-tubulin and normalized to K562 AAVS1KO (control is set to one). Data show mean ± SEM from six independent experiments. (B) Representative proliferation assay and quantitative analysis of mean cell growth doubling time ± SEM (C) for K562 AAVS1KO (*n* = 8) and K562 UCP2 KO single cell colonies (K562 UCP2 KO Oligo1 colony1 (O1C1) (n = 8), K562 UCP2 KO Oligo1 colony7 (O1C7) (*n* = 4), K562 UCP2 KO Oligo2 colony3 (O2C3) (*n* = 3) and UCP2 KO Oligo2 colony9 (O2C9) (*n* = 5). (D) Representative changes in extracellular acidification rate (ECAR) and (E) oxygen consumption rate (OCR) during a mito stress assay. (F–I) ECAR (F), OCR (G), spare respiratory capacity (H) and coupling efficiency (I) alterations in K562 UCP2 KO single cell colonies compared to K562 AAVS1 KO. Data show mean ± SEM of three independent experiments. Oligo, oligomycin; A/R, antimycin and rotenone.

As a next step, we knocked out UCP2 in K562 cells using the CRISPR-Cas9 approach and obtained 4 clones with validated UCP2 KO (Fig. 4A). Consistent with our previous data, the proliferation rate remained unchanged between UCP2 KO and control KO cells (K562 AAVS1 KO) (Fig. 4B and C).

Supportive metabolic flux analysis showed no changes upon UCP2 KO in K562 cells (Fig. 4D-I). Notably we observed variation between different single cell colonies (Fig. 4G), demonstrating the importance of the broad panel of controls we used.

**Fig. 5:**
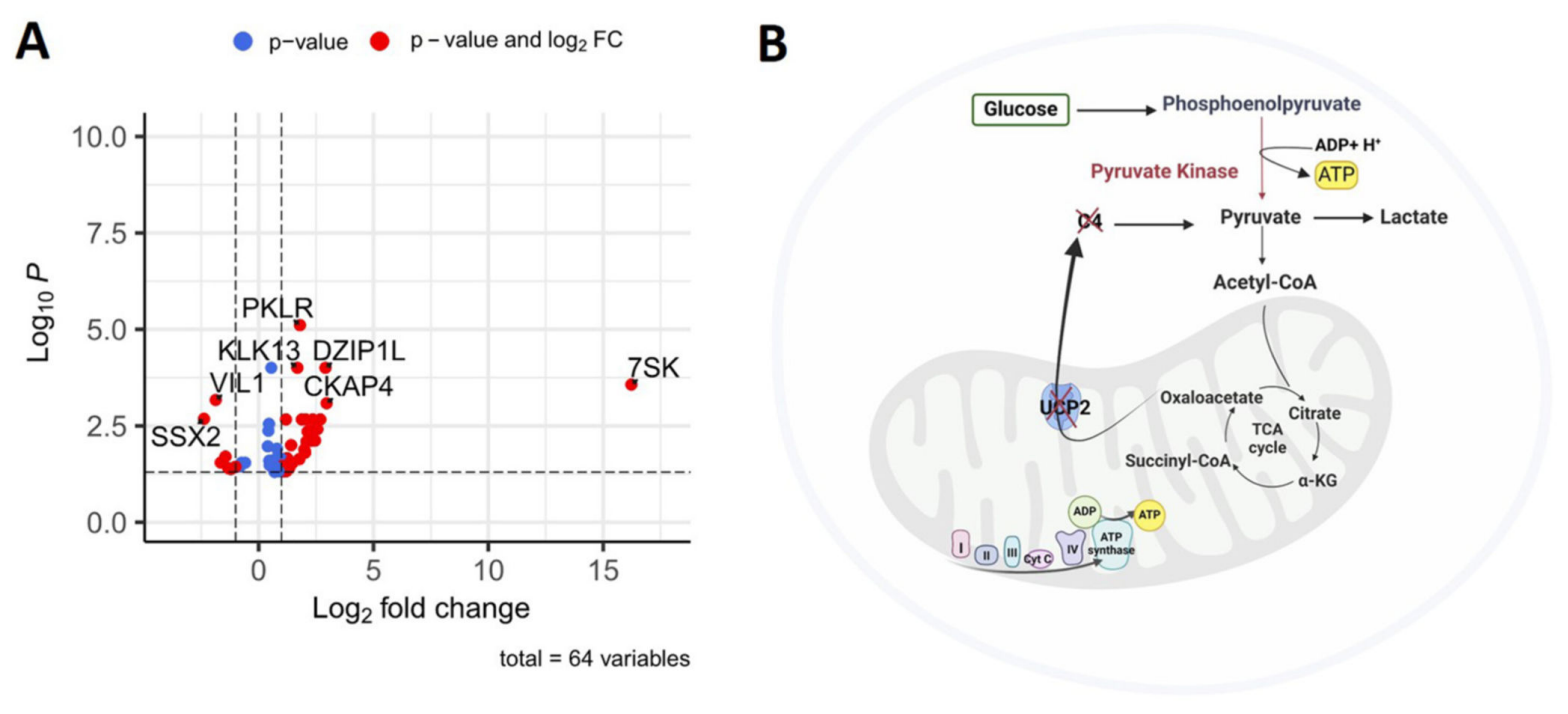
UCP2 depletion increases the PKRL gene activity (A) a volcano plot of UCP2 KO RNA sequencing entities compared to K562 AAVS1 KO cells. (B) K562 UCP2 KO cells upregulate pyruvate kinase (PKRL) to compensate for the reduced pyruvate and ATP production caused by the absence of UCP2 to enable the transport of C4 metabolites from the mitochondria, created with BioRender.com.

To gain a better understanding of any molecular changes in the K562 UCP2 KO cells, we performed RNA sequencing and compared all entities with high gene activity alterations in all UCP2 KO clones compared to control KO cells (Fig. 5A). Interestingly, among the few entities with significant changes in UCP2 KO cells, pyruvate kinase R/L (*PKRL*) showed the highest gene expression changes. *PKRL* encoded the pyruvate kinase that is responsible for the conversion of phosphoenolpyruvate (PEP) to pyruvate, a rate limiting step of glycolysis. The upregulation of *PKRL* may compensate for the loss of UCP2 to maintain a high glycolytic metabolism, as demonstrated in Fig. 5B.

## 4 Discussion

Using a wide range of cancer cell types from both mice and humans, we show that although UCP2 is expressed in all cancer cells, the abundance of UCP2 varies between different cancers. The cancer cell response to glucose and glutamine deprivation did not uniformly correlate with UCP2 abundance and cell proliferation. Additionally, we evaluated the effect of UCP2 depletion in one cell line. Depletion per se did not alter proliferation rate or cell metabolic state. Despite the observed divergence in basal UCP2 protein levels and their changes upon glucose and glutamine deprivation, hypoxic conditions consistently resulted in significant reduction of UCP2.

The complexity of cancer metabolism and the ability of cancer cells to adapt to various environmental stressors represent a major challenge for oncologists. UCP2 is currently regarded as a pivotal factor of metabolic flexibility, implicated either in the modulation of ROS levels [38,39] or in the translocation of C4 metabolites [12,13,16,40]. However, the current literature about the role of UCP2 in cancer is controversial. Yu et al. reported that higher expression levels of UCP2 correlated with worse clinical characteristics in gallbladder cancer [41]. Conversely, other studies observed increased colon tumorigenesis in UCP2 knockout mouse models, potentially due to disruptions in lipid metabolism and NADPH homeostasis [42,43]. The research of Zhao et al. [44] delineates the role of UCP2 in oncogenic activation and proliferation, thereby underscoring its contribution to the adaptability and survival of cancer cells [45,46].

Our investigation challenges the initial hypothesis that UCP2 uniformly underpins cancer cell survival under conditions of nutrient deprivation. Instead, it reveals a heterogeneity in UCP2 expression and its regulatory mechanisms. This diversity accentuates the complexity of metabolic adaptation within cancerous tissues. Our observations suggest that the role of UCP2 in cancer metabolism is not universal across all cancer types. It should also be mentioned that the experiments were performed in vitro, and therefore all interactions that tumors may be exposed to in vivo (nutrient supply, irrigation, pH, …) are not present. All these could explain the controversial results obtained in studies with different cell lines. Additionally, this finding highlights the intricate regulatory mechanisms of UCP2, which appear contingent upon the specific metabolic requirements and the microenvironmental context of each cancer cell type. Our study provides further evidence to support previous findings on the regulatory complexity of UCP2, particularly in the context of glucose deprivation and hypoxia [16,46].

The swift downregulation of UCP2 following CoCl2-induced hypoxia mimicking conditions, without concurrent metabolic alterations, suggests an adaptive response rather than a direct reaction to changes in metabolic states. This adaptation likely represents a broader survival strategy, modulating UCP2 expression to sustain metabolic flexibility amidst environmental fluctuations. Furthermore, our findings resonate with perspectives that advocate for a broader functional purview of UCP2 beyond metabolism alone.

The lack of significant metabolic alterations upon UCP2 knockout, as demonstrated in our CRISPR-Cas9 K562 cell line model, challenges prevailing views on UCP2’s metabolic role. This suggests that UCP2’s function may be complemented by other mitochondrial proteins, forming a complex regulatory network rather than acting as a solitary modulator of cancer cell metabolism. The question of which proteins may compensate for UCP2’s loss in C4 metabolite transport remains open. Intriguingly, our RNA-seq analysis revealed a marked increase in pyruvate kinase transcription in UCP2 KO K562 cells. This enzyme, which is crucial for glycolysis by catalyzing the conversion of phosphoenolpyruvate (PEP) to pyruvate, suggests a strategic cellular adaptation to maintain glycolytic flux in the absence of UCP2 [47]. This highlights the adaptive capacity of cancer cells to uphold metabolic demands despite potential disruptions in mitochondrial function. The critical role of pyruvate kinase, particularly its M2 isoform (PKM2), in cancer metabolism and as a therapeutic target is well-established. PKM2’s involvement in redirecting glucose metabolism towards lactate production and its regulatory role at the transcriptional level are pivotal in tumorigenesis and cancer cell metabolism [48]. Moreover, the overexpression of PKM2 in various cancers, its link to tumor proliferation and metastasis, and its regulatory interactions within the tumor microenvironment and with lncRNAs underscore the enzyme’s complexity and potential as both a diagnostic marker and therapeutic target [49,50].

In conclusion, our findings demonstrate that UCP2 plays a more complex, context-dependent role in cancer cell metabolism and survival. A comprehensive understanding of UCP2 role within the broader context of cancer metabolism will help by the development of targeted therapies capable of disrupting the metabolic flexibility essential for cancer cell survival.

## Supplementary Material

**Appendix A. Supplementary data**

Supplementary data to this article can be found online at https://doi.org/10.1016/j.bbabio.2024.149486.

Supplementary Material

## Data Availability

Data will be made available on request.
