# Supplementary material for: Differential regulation of mitochondrial uncoupling protein 2 in cancer cells
Source: Biochim Biophys Acta Bioenerg. Author manuscript; Available in PMC 2025 May 9. (PMC7617651; doi:10.1016/j.bbabio.2024.149486)
Supplement: Supplementary Material [file EMS204737-supplement-Supplementary_Material.pdf]

Supplementary figures

Differential regulation of mitochondrial uncoupling protein 2 in cancer cells

Taraneh Beikbaghban[1], Ludovica Proietti[2], Jessica Ebner[2], Roko Sango[3,4,5], Thomas Rattei[3], Thomas Weichhart[6], Florian Grebien[2,7,8], Felix Sternberg[1,#,*], Elena E. Pohl[1,*]

[1] Institute of Physiology, Pathophysiology and Biophysics, University of Veterinary Medicine, Vienna, Austria

[2] Institute for Medical Biochemistry, University of Veterinary Medicine, Vienna, Austria

[3] Centre for Microbiology and Environmental Systems Science, University of Vienna, Vienna, Austria

[4] Center of Pathobiochemistry and Genetics, Institute of Medical Genetics, Medical University of Vienna, Vienna, Austria

[5] Doctoral School in Microbiology and Environmental Science, University of Vienna, Vienna, Austria

[6] Center of Pathobiochemistry and Genetics, Institute of Medical Genetics, Medical University of Vienna, Austria.

[7] St. Anna Children's Cancer Research Institute (CCRI), Vienna, Austria

[8] CeMM Research Center for Molecular Medicine of the Austrian Academy of Sciences, Vienna, Austria

[#] Present address: Department of Nutritional Sciences, Faculty of Life Sciences, University of Vienna, Austria.

* Corresponding authors: elena.pohl@vetmeduni.ac.at, felix.sternberg@univie.ac.at

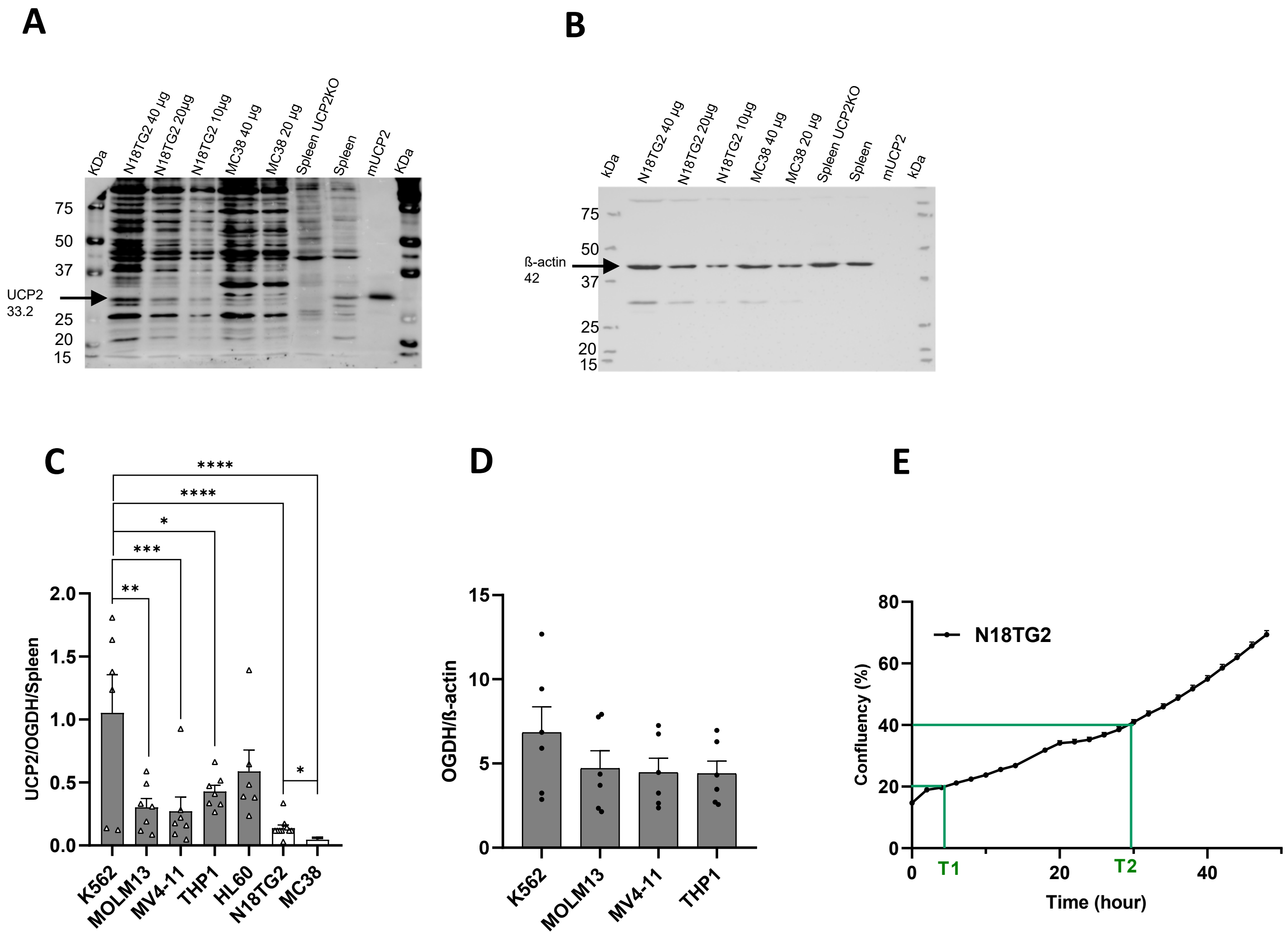

Supplementary Figure 1. UCP2 is differentially expressed in cancer cells. (A-B) Representative Western blot analysis of UCP2 (A) and ß-actin (B) expression in mouse cancer cell lines (MC38 and N18TG2). (C) Quantification of UCP2 levels in human (K562, MOLM13, MV4-11, THP1 and HL60) and mouse (MC38 and N18TG2) cancer cell lines. UCP2 protein levels were normalized to OGDH levels and 20 µg mouse spleen were used as an internal control. Data are presented as mean ± SEM of six independent experiments. (D) Quantification of OGDH expression in human cancer cells (K562, MOLM13, MV4-11 and HTP1). OGDH protein levels are represented as normalized to ß-actin. Data are presented as mean ± SEM of five independent experiments. (E) Representative proliferation assay of N18TG2 cells grown for 48 hours. The corresponding time points to 20% and 40% confluency are marked as T1 and T2, respectively. The time required for N18TG2 cells to double their confluency was reported as the doubling time (ΔT= T2-T1).

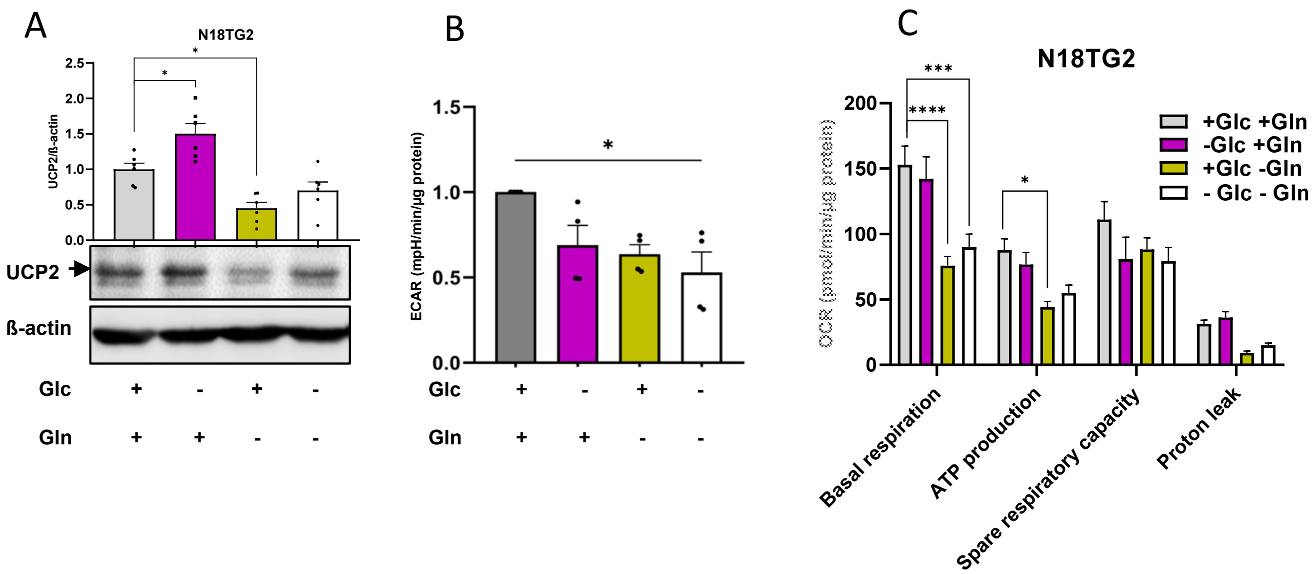

Supplementary Figure 2. Changes in UCP2 and cell metabolism upon glucose and glutamine starvation in N18TG2 cells (A) Representative Western blot analysis and the corresponding quantification of UCP2 and ß-actin expression in N18TG2 cells after 4 h of glucose, glutamine, both glucose (Glc) and glutamine (Gln) starvation compared to control (+Glc +Gln). The amount of protein is presented as the ratio between the band intensities of UCP2 and ß-actin and normalized to the band intensity of cells grown under control conditions (control is set to one). Data are presented as mean ± SEM of six independent experiments. (B-C) Quantification of the basal extracellular acidification rate (ECAR) (B), basal oxygen consumption rate (OCR), ATP-linked OCR, spare capacity and proton leak (C) in N18TG2 cells under glucose and glutamine starvation for 4 h compared to control. Data are presented as mean ± SEM of four independent experiments

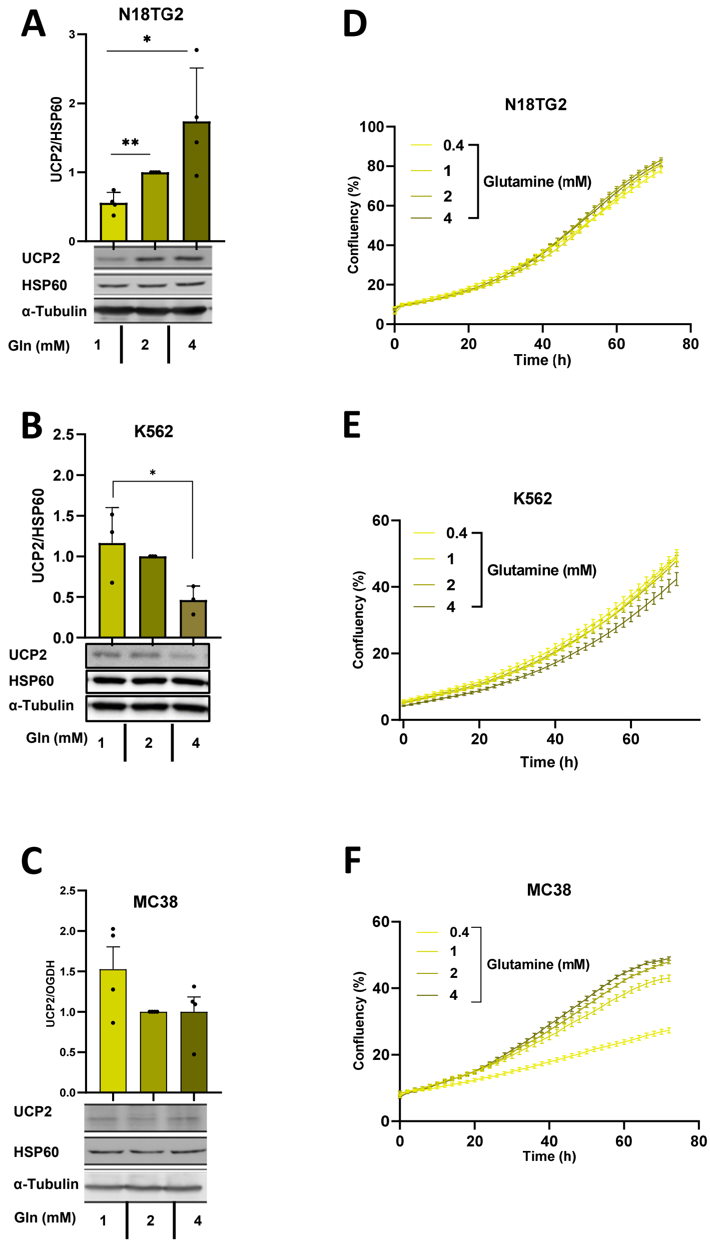

Supplementary Figure 3. UCP2 and cell proliferation changes upon glutamine deprivation. (A-C) Representative Western blot analysis and corresponding quantification of UCP2 and HSP60 expression changes upon glutamine (Gln) concentration variations in N18TG2 (A), K562 (B) and MC38 (C), respectively. The amount of protein is presented as the ratio between the band intensities of UCP2 and HSP60 and normalized to the band intensity of cells grown in 2 mM Gln as control (control is set to one). (D-F) Effect of glutamine deprivation on the proliferation rate of N18TG2 (D), K562 (E) and MC38 (F) over 72 hours. Data are presented as mean ± standard deviation of three independent experiments.
